# Aging Contributes to Inflammation in Upper Extremity Tendons and Declines in Forelimb Agility in a Rat Model of Upper Extremity Overuse

**DOI:** 10.1371/journal.pone.0046954

**Published:** 2012-10-03

**Authors:** David M. Kietrys, Ann E. Barr-Gillespie, Mamta Amin, Christine K. Wade, Steve N. Popoff, Mary F. Barbe

**Affiliations:** 1 Department of Rehabilitation and Movement Sciences, University of Medicine and Dentistry of New Jersey, School of Health Related Professions, Stratford, New Jersey, United States of America; 2 College of Health Professions, Pacific University, Hillsboro, Oregon, United States of America; 3 Department of Anatomy and Cell Biology, Temple University School of Medicine, Philadelphia, Pennsylvania, United States of America; 4 Department of Physical Therapy, Thomas Jefferson University, Philadelphia, Pennsylvania, United States of America; St. Jude Children's Research Hospital, United States of America

## Abstract

We sought to determine if tendon inflammatory and histopathological responses increase in aged rats compared to young rats performing a voluntary upper extremity repetitive task, and if these changes are associated with motor declines. Ninety-six female Sprague-Dawley rats were used in the rat model of upper extremity overuse: 67 aged and 29 young adult rats. After a training period of 4 weeks, task rats performed a voluntary high repetition low force (HRLF) handle-pulling task for 2 hrs/day, 3 days/wk for up to 12 weeks. Upper extremity motor function was assessed, as were inflammatory and histomorphological changes in flexor digitorum and supraspinatus tendons. The percentage of successful reaches improved in young adult HRLF rats, but not in aged HRLF rats. Forelimb agility decreased transiently in young adult HRLF rats, but persistently in aged HRLF rats. HRLF task performance for 12 weeks lead to increased IL-1beta and IL-6 in flexor digitorum tendons of aged HRLF rats, compared to aged normal control (NC) as well as young adult HRLF rats. In contrast, TNF-alpha increased more in flexor digitorum tendons of young adult 12-week HRLF rats than in aged HRLF rats. Vascularity and collagen fibril organization were not affected by task performance in flexor digitorum tendons of either age group, although cellularity increased in both. By week 12 of HRLF task performance, vascularity and cellularity increased in the supraspinatus tendons of only aged rats. The increased cellularity was due to increased macrophages and connective tissue growth factor (CTGF)-immunoreactive fibroblasts in the peritendon. In conclusion, aged rat tendons were overall more affected by the HRLF task than young adult tendons, particularly supraspinatus tendons. Greater inflammatory changes in aged HRLF rat tendons were observed, increases associated temporally with decreased forelimb agility and lack of improvement in task success.

## Introduction

Upper extremity work-related musculoskeletal disorders (WMSDs) are common and economically burdensome, and accounted for 29 percent of all workplace injuries and illnesses in the U.S. requiring time away from work in 2010 [1]. Epidemiological evidence suggests that several factors play a role in these disorders, including physical, mechanical, and individual predisposing factors, such as age, gender and lifestyle [2], [3], [4]. Studies indicate that older workers may be more susceptible to WMSDs than younger workers because of decreased physical capacity or a greater propensity for injury [5], [6], [7]. Our work and others have found a link between inflammation and decreased sensorimotor function in patients with WMSDs and in our rat model of WMSDs, although these studies were performed in young adult patients or rats [8], [9], [10], [11]. The Health and Safety Executive Laboratory has called for more work on the contribution of aging to WMSDs [12].

In a rat model of upper extremity WMSDs, we found that aging enhanced a pro-inflammatory serum cytokine response that was greater in aged rats performing a high repetition low force (HRLF) task, compared to young adult HRLF rats [13]. Inflammatory cytokines also increased in spinal cord neurons in aged rats with overuse-induced peripheral neuropathy, an increase associated with forepaw tactile hypersensitivity and decreased grip strength [14]. The combination of aging and HRLF task performance did not lead to greater declines in grip strength than that found in young adult HRLF rats [13]. However, we have not examined for changes in other upper extremity motor skills. We speculate that increased tissue inflammatory cytokines occurring as a consequence of both aging and repetitive task performance will contribute to declines in motor abilities and tissue pathology.

Shoulder subacromial impingement syndrome is associated with work-related factors, such as force requirements, lifting, repetitive shoulder or wrist/hand movement, hand-arm vibration, and posture (working with the arm above shoulder level) [15]. In addition, a recent prospective population-based study found occupational physical loading (including repetitive movement, vibration, lifting and working in awkward postures) were significant predictors of subsequent shoulder disorders [16]. Tendinopathies of the hand and wrist are also associated with performance of repetitive, forceful tasks in the workplace [4], [17], [18]. Inflammatory cytokines are implicated in the evolution of tendon pathology and play a role in oxidative stress-induced cellular apoptosis [19], [20], [21]. Several animal studies have reported that prolonged downhill or treadmill running induces inflammation and injury in flexor digitorum and supraspinatus tendons, including increased cellularity and collagen disorganization [22], [23], [24], [25], [26], [27], [28]. Age-related changes have also been reported in the supraspinatus tendon and other subacromial tissues [29], [30], [31], although to our knowledge, only one study to date has examined the combined effects of occupational risk factors and aging on the shoulder disorders [16]. Miranda et al found that lifting was a strong predictor of subsequent shoulder disorders in older individuals [16].

Therefore, here, we examined the effects of performing a high repetition low force (HRLF) handle-pulling task for 12 weeks on several attributes of upper extremity motor function and tendon pathophysiology in aged and young adult rats. Our first aim was to examine the effects of this task on reach performance (reach rate, the percentage of successful reaches and grasp phase time), and forelimb agility. Our second aim was to examine for task-induced increases in inflammation and histopathology in flexor digitorum and supraspinatus tendons of aged and young adult HRLF rats. We hypothesized that aged HRLF rats would show greater declines in motor function, and that, although both tendons would show evidence of pathology, the aged HRLF rat tendons would show greater signs of inflammation and pathology. We further hypothesized that the motor declines would be temporally associated with tendon inflammation and injury.

## Methods

### Subjects

This study was carried out in strict accordance with the recommendations in the Guide for the Care and Use of Laboratory Animals of the National Institutes of Health. The protocol was approved by the Temple University Institutional Animal Care and Use Committee. All surgery was performed under sodium pentobarbital anesthesia, and all efforts were made to minimize suffering. Adult female rats were used for several reasons: (1) Human females have a higher incidence of work-related MSS/MSDs than males [32]; (2) for comparison purposes to our past studies on females; (3) and, the examination of male rats, which are both larger and stronger, would require adjustments in operant conditioning equipment and conditions, including a switch to higher capacity force transducers, as ours were chosen for their sensitivity to the force generating capabilities of adult female rats. Rats were housed in a central animal facility in separate cages with a 12-hour light: dark cycle and free access to water. They were weighed weekly and their food was adjusted to maintain 95% body weight of age-matched controls.

A total of 67 aged (14–18 months of age) and 29 young adult (2.5 to 6.5 months of age) female Sprague-Dawley rats were used (96 total rats). Forty-nine aged rats and 14 young adult rats were randomly assigned to the task groups, and then were food restricted to within ±5% of normal control (NC) weight and trained to perform a high repetition, low force (HRLF) reaching and handle-pulling task, for 10 minutes/day, 5 days/week, for 4 weeks. These rats then performed this HRLF task for 2 hrs/day, in 30 min sessions that were separated by 90 min breaks, 3 days/wk, for up to 12 weeks. Due to euthanasia for tissue collection, the number per weekly endpoint declined in the aged HRLF rats across weeks of task performance: 3 weeks (n = 49 aged), 6 weeks (n = 43 aged), 9 weeks (n = 34 aged), or 12 weeks (n = 16 aged). Fourteen young adult HRLF rats were not euthanized until the 12-week endpoint; another four were euthanized for histological assay at week 6. An additional 18 aged rats and 11 young adult rats were randomly assigned to serve as age-matched normal controls (NC) with free access to food. The NC rats did not undergo training or task performance. Lastly, 10 aged rats were eliminated from the study due to renal failure, presence of tumors or mortality; these were not included in the counts described above. Many of these losses occurring in the most elderly rats, as the 18 month time point was reached, creating lower n numbers for 12 week aged rats than 9 week aged rats.

### Behavioral Apparatus and Task

The custom designed behavioral apparatuses used in this study have been previously described [9], [33], [34]. Briefly, animals reached through a shoulder height portal and then isometrically pulled a handle. If the animal met the required force (15% ±2.5% of maximum pulling force) and time (50 ms) criteria, and reached at a rate of 4 reaches or more/min, a food pellet reward was dispensed. In order to maintain their interest in food pellets, task rats remained food-restricted to no more than 5% less than age-matched NC rats. Following a 4-week training period, task rats performed the HRLF task for up to 12 weeks. Animals were allowed to use their preferred limb to reach, and the contralateral limb as support [35]. Data was recorded from the preferred (reach) limb.

### Determination of Reach Performance Behaviors

Force lever data were used to calculate reach rate (all reaches/min), the percentage of successful reaches, and grasp phase time, as previously described [9], [36]. The force lever data were obtained from randomly selected subsets of 17 aged HRLF rats in weeks 1 and 9, and 8 animals in week 12 of task performance; and from 14 young adult HRLF rats in weeks 1, 9 and 12 of task performance. The subset sample sizes were based on a priori sample size estimates to achieve 80% statistical power. Force lever data could not be collected from normal control rats, as they did not perform the task.

### Determination of Forelimb Agility

The forehead sticker removal (FHSR) [37] test was used to determine functional agility of the forelimb. This motor grooming skill was determined at the end of weeks 1, 3 and 12 in 16 aged HRLF rats, 18 aged NC rats, 14 young adult HRLF rats, and 8 young adult NC rats. Rats were graded on a 6-point scale (0 = no attempt at sticker removal to 5 = successful removal of sticker) with either the right or left forearm [37]. The test was repeated 5 times per time point, and the best score for each limb was recorded individually.

### Tendon Cytokine Analysis

For this analysis, forelimb flexor digitorum tendons were collected from 15 aged HRLF rats at 3 (n = 3), 6 (n = 4), 9 (n = 7) or 12 (n = 5) weeks after HRLF task onset (i.e., at 15.75 to 18 months of age at time of tissue collection), and from 6 aged NC rats (18 months of age). These tendons were also collected from 5 young adult HRLF rats at 12 weeks after HRLF task onset (i.e., at 6.5 months of age at time of tissue collection, and from 5 young NC rats (6.5 months of age). Rats were first euthanized with an overdose of sodium pentobarbital (Nembutal; 120 mg/kg body weight), tendons collected, homogenized and analyzed for interleukin 1 beta (IL-beta), IL-6, IL-10, and tumor necrosis factor alpha (TNF-alpha) using commercially available ELISA kits as previously described [38].

### Tendon Histomorphometry and Immunohistochemistry

Tissues for this analysis were collected from 21 aged HRLF rats at 6 (n = 4), 9 (n = 10) or 12 (n = 4) weeks after onset of HRLF task performance, 12 young adult HRLF rats at either 6 weeks (n = 4) or 12 weeks (n = 8) after onset of HRLF task performance, 6 aged NC rats (18 months of age), and 5 young adult NC rats (6.5 months of age). Rats were euthanized with sodium pentobarbital (120 mg/kg body weight), and perfused transcardially with 4% paraformaldehyde in 0.1 M PO_4_ buffer (pH 7.4). Forelimb flexor digitorum and supraspinatus tendons were removed from the bones, immersion fixed for several days, then equilibrated in 30% sucrose in phosphate buffer for 2 days before cyrosectioning into 12 µm longitudinal sections. Sections were mounted onto charged slides and stored at −80C until use. The supraspinatus enthesis was prepared for examination by decalcifying the shoulder (humerus + scapula), embedding them in paraffin and sectioned, as previously described [39]. The remaining 5 aged 12-wk HRLF and 5 aged NC shoulder samples were embedded without decalcification in methyl methacrylate, as described previously. These samples were cut into 5 µm anterior-posterior sections using a diamond saw, mounted onto slides, and stained with von Kossa in order to assay for tendon calcification. A selection of frozen sectioned supraspinatus samples of each age group were also stained with von Kossa.

For histomorphometric analysis, the sections were dried stained with hematoxylin and eosin (H&E). Both preferred and reach limb were examined. Tendons were scored in a blinded manner by three examiners (DK, MA, MB) using a modified Bonar scale [26], [35], [40]. We assessed overall cellularity, the organization of collagen fibrils, and vascularity, with cellularity further quantified using an image analysis system, as described below. For each category of the Bonar scale, a normal appearance was assigned a score of 0. Pathological changes were scored from 1 to 3, with 3 representing advanced pathological changes. For supraspinatus tendon, sections were scored in 3 different locations using a 300 and 600 × magnification: 1) the enthesis, 2) the cut distal end of the tendon near the enthesis but entering the muscle, and 3) the mid-substance of the tendon approximately 2 mm proximal to the distal end (entirely intramuscular). Forelimb flexor digitorum tendons were examined at wrist level.

Adjacent sections of supraspinatus and forelimb flexor digitorum tendons were immunostained for anti-CD68 antibody (ED1)-positive macrophages or connective tissue growth factor (CTGF)-immunoreactive cells, using previously described methods [35], [41]. The numbers of ED1-IR macrophages were counted as previously described [41], in 3 adjacent fields per tissue and per region. CTGF immunostaining was quantified as a percent area of pixels in the selected field with immunostaining, as described previously [35].

### Data Analysis

All statistical tests were performed using PRISM 5 (GraphPad Software, Inc., San Diego, CA). A p value of <0.05 was considered significant. Percentage of successful reaches and grasp phase time data was analyzed using 2-way ANOVA, with age and week as factors. Forehead sticker removal (FHSR) scores were analyzed with two-way ANOVA, with limb and week as factors, and then secondarily by age using two-tailed t-tests between matching weeks of task performance (p values were adjusted to take into account the multiple comparisons), as were inflammatory cytokine levels and ED1-IR cell counts. For forelimb flexor digitorum tendons, histomorphometric data from both preferred limb and support limb was obtained and showed no differences between limbs. Therefore, morphology data from tendons of both limbs was combined, and the variables analyzed via one-way ANOVA using week as the factor. Supraspinatus histomorphometry scores and ED1-IR macrophage data from the preferred reach limbs were analyzed with one-way ANOVAs. For each ANOVA, the Bonferroni method for multiple post hoc comparisons was used, and adjusted p values are reported.

## Results

### Reach Performance

**Figure 1:**
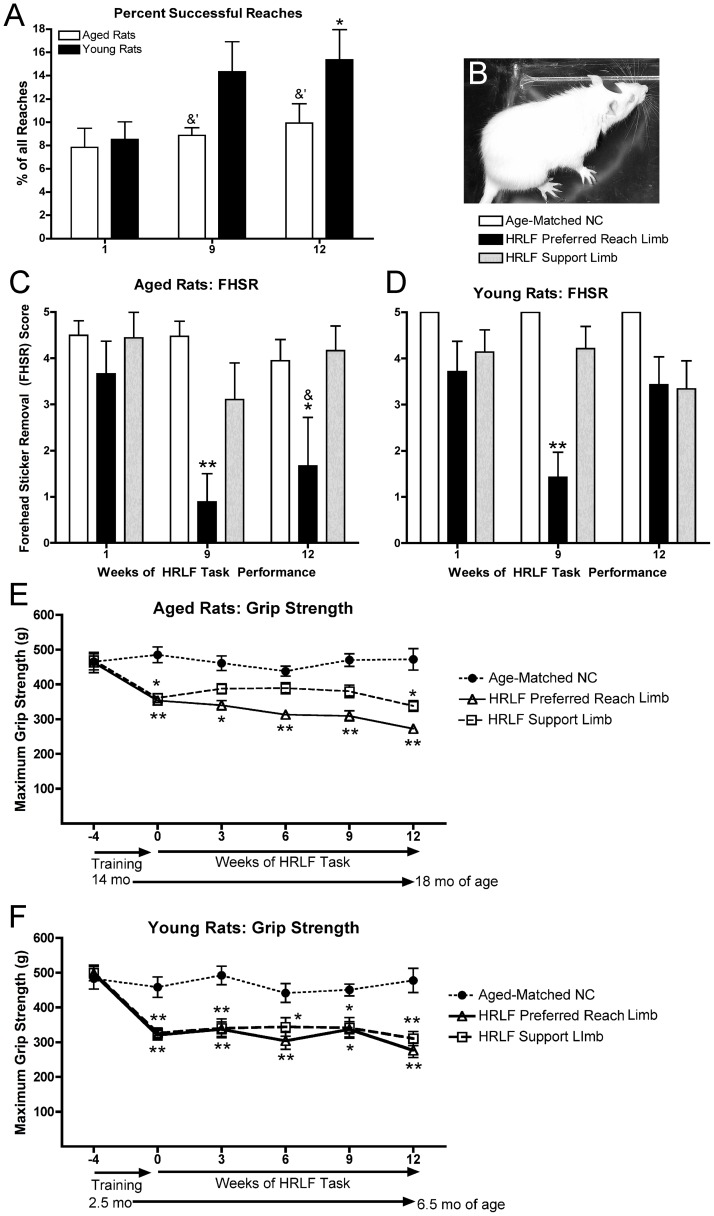
Motor performance changes in aged and young adult rats with high repetition low force (HRLF) task performance. *(A)* Percentage of successful reaches. *(B)* A rat during the forehead sticker removal test. *(C)* Forehead sticker removal score (FHSR; 0 = no attempt at sticker removal to 5 = successful removal of sticker) with preferred reach or support forelimb. *(D)* FHSR in young adult HRLF and NC rats *: p<0.05, **: p<0.01, compared to aged-matched NC; &: p<0.05, compared to the same week in the other age group.

The percentage of successful reaches was lower in aged rats than in young adult HRLF rats in weeks 9 and 12 (Fig. 1A). Aged HRLF rats showed no differences with task performance, but young adult HRLF rats had a significant increase in the percentage of successful reaches in week 12, compared to their week 1 (p<0.05). No significant differences were observed in grasp phase time with task performance in either age group (data not shown), nor were there changes in reach rate in aged HRLF rats with task performance (data not shown).

### Forelimb Agility

Both aged and young adult HRLF rats had significantly decreased ability to remove a sticker placed on their forehead (Fig. 1B–D). Post hoc analysis showed significant declines in preferred reach limbs of aged rats in weeks 9 and 12 (p<0.01 and p<0.05, respectively; Fig. 1C), and in preferred reach limbs of young adult rats in week 9 (p<0.01; Fig. 1D), compared to week 1. Improvement was seen in young HRLF rats by week 12 (Fig. 1D). FHSR scores were lower in aged in their preferred reach limbs by week 12, compared to young adult rats (p = 0.003) (Fig. 1C,D).

### Inflammatory Cytokines in Flexor Digitorum Tendons are Affected by Age and Task Performance

**Figure 2:**
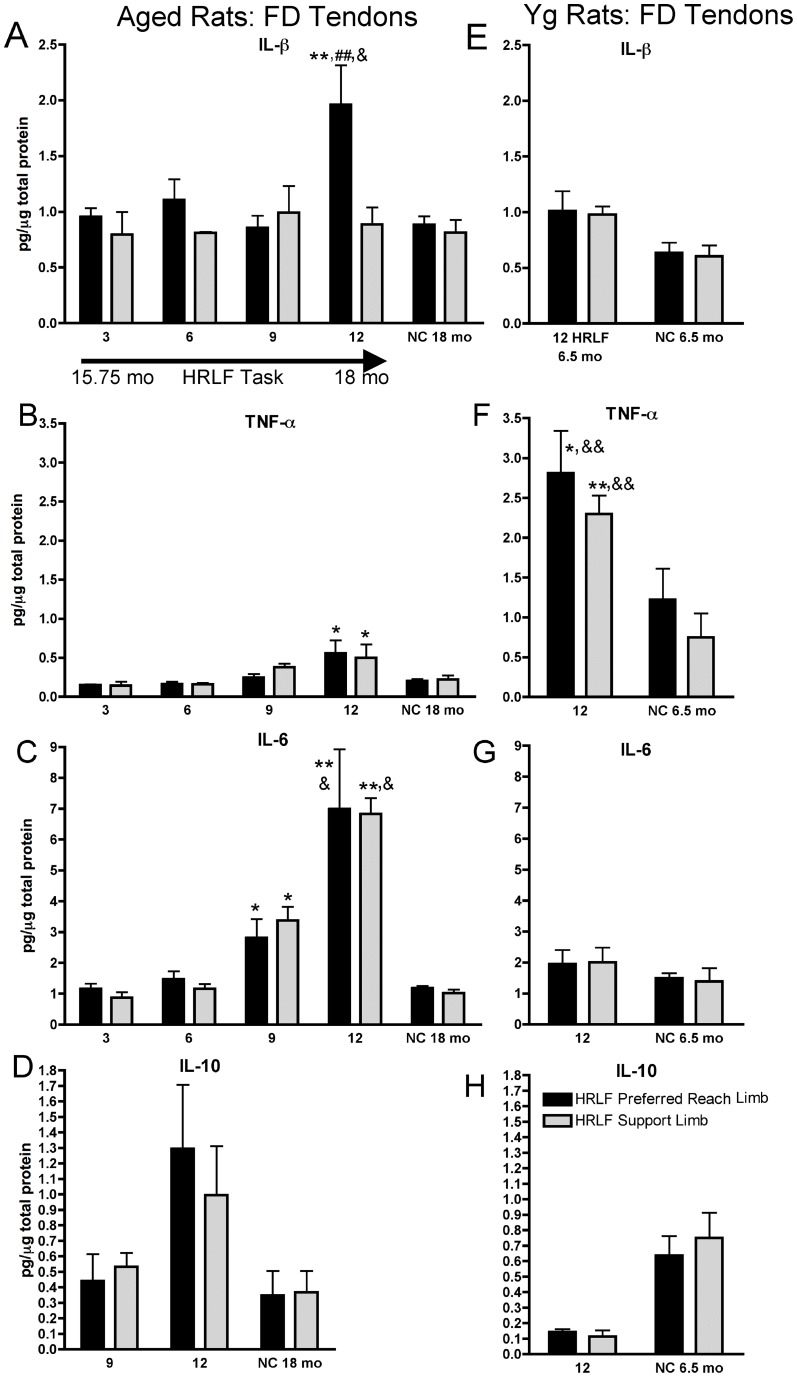
Pro-inflammatory cytokine levels in aged and young rat forelimb flexor digitorum tendons. *(A–D)* IL1-β, TNF-α, IL-6 and IL-10 in aged rats’ preferred reach limb and support limb tendons. *(E–H)* IL1-β, TNF-α, IL-6 and IL-10 in young adult rat tendons. *: p<0.05 and **: p<0.01, compared to aged-matched NC rats; ##:p<0.01 compared to support limb of same-aged 12 week HRLF rats: &: p<0.05 and &&: p<0.01, compared to same week and limb of other age group.

In aged rats, ELISA assessed IL1-β increased in flexor digitorum tendons of preferred reach limbs of 12-week HRLF rats, compared to age-matched NC (p<0.01), and compared to their contralateral support limb (p<0.01; Fig. 2A). TNF-alpha increased, bilaterally, in 12-week HRLF aged rats, compared to NC (p<0.05 each; Fig. 2B). IL-6 increased, bilaterally, in 9-week and 12-week HRLF aged rats, compared to NC (p<0.05 each; Fig. 2C). In young adult rats, IL-1beta and IL-10 did not increase with task performance (Fig. 2E,G,H). In contrast, TNF-alpha levels increased, bilaterally, in flexor digitorum tendons of young adult 12-week HRLF rats, compared to age-matched NC (p<0.05 and p<0.01 in reach and support limbs, respectively; Fig. 2F). There were no significant increases in IL-10 in either age group (Fig. 2D,H).

Inflammatory cytokines in flexor digitorum tendons showed age-related differences (Fig. 2A, C, F). IL-1beta increased in preferred reach limb tendons of 12-week HRLF aged rats, compared to 12-week HRLF young adult rats (p<0.05; Fig. 2A,E). IL-6 increased, bilaterally, in tendons of 12-week HRLF aged rats, compared to young adult rats (p<0.05 each; Fig. 2C,G). The reverse was true for TNF-alpha, which was higher, bilaterally, in 12-week HRLF young adult rat tendons (p<0.01 each; Fig. 2B,F).

### Histomorphological Changes: CTGF+ and ED1+ Cells Increase with Task and Age

**Figure 3:**
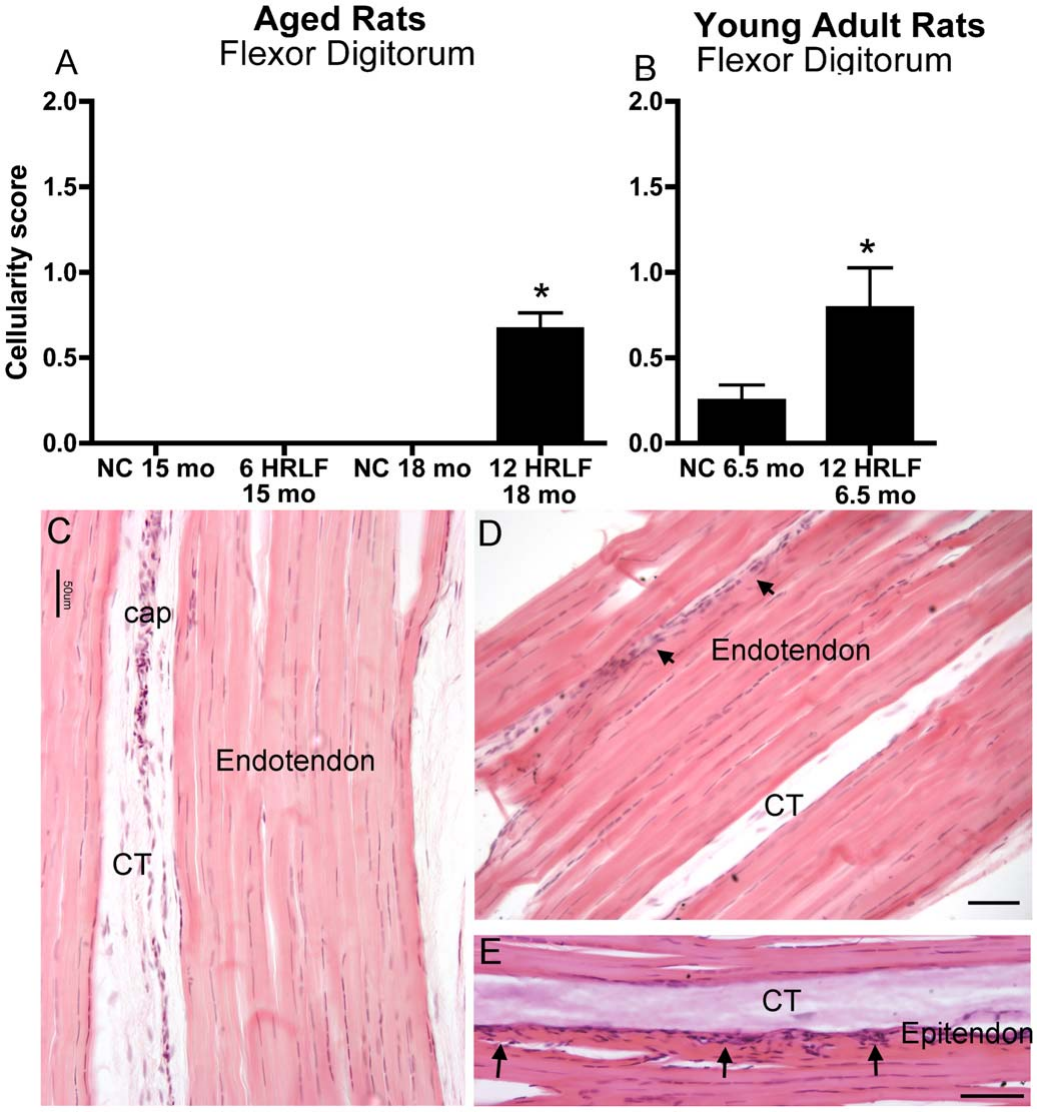
Forelimb flexor digitorum tendons at wrist level of NC and HRLF rats. *(A,B)* Cellularity scores in normal control (NC) and high repetition low force (HRLF) aged and young adult rats. *:p<0.05, compared to age-matched NC rats. *(C)* Photo of a flexor digitorum tendon (T) at wrist level from a young NC rat. Cap = capillary; CT = connective tissue. *(D & E)* Photos of flexor digitorum tendons (T) at wrist level from two different young 12-week HRLF rats. Arrows in D and E indicate sites of increased cellularity in the epitendon. Scale bar = 50 µm.

When quantified with the Bonar scale, cellularity increased in flexor digitorum tendons of 12-week HRLF rats of both age groups, compared to age-matched NC (p<0.05 each; Fig. 3A,B). Fig. 3E shows increased cellularity within the epitendon of a young adult HRLF rat. However, there were no statistical differences in endotendon collagen organization or vascularity in flexor digitorum tendons with task performance of either age group (Fig. 3C,D).

**Figure 4:**
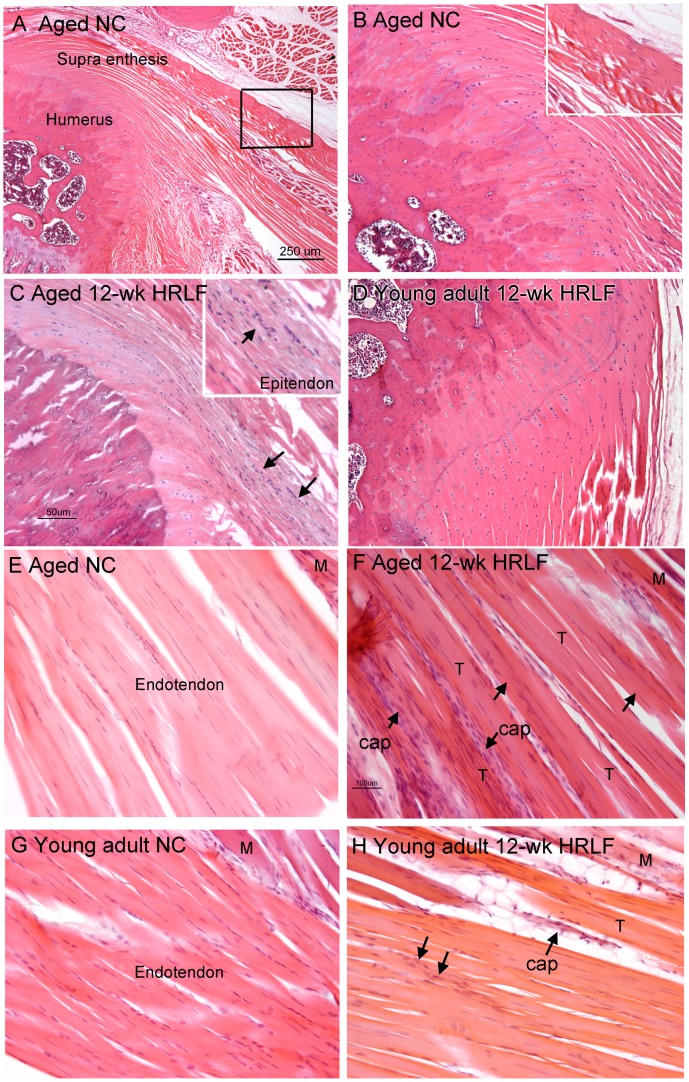
Supraspinatus tendons of NC and HRLF rats. *(A)* A low power photo of the enthesis of a supraspinatus tendon attaching to greater tuberosity of humerus in an aged NC rat. Scale bar = 250 µm. *(B)* A higher power image of same section as shown in A. Inset is an enlargement of boxed area shown in A. *(C&D).* Photos of the enthesis of supraspinatus tendons in *(C)* an aged 12-week HRLF rat, and *(D)* a young adult 12-week HRLF rat. Arrows in C indicate increased site of cellularity, a region shown enlarged in inset. Scale bar in C is 50 µm; panels B and D are at same magnification. (E–H) Photos of distal cut ends of a supraspinatus tendon in: *(E)* an aged NC rat, *(F)* aged 12-week HRLF rat, *(G)* young adult NC rat, and *(H)* young adult 12-week HRLF rat. Arrows in F and H point out sites of increased cellularity and vascularity (cap = capillary bed). T = tendon; M = muscle fibers. Scale bar in F is 100 µm; panels E, G and H are at same magnification.

**Figure 5:**
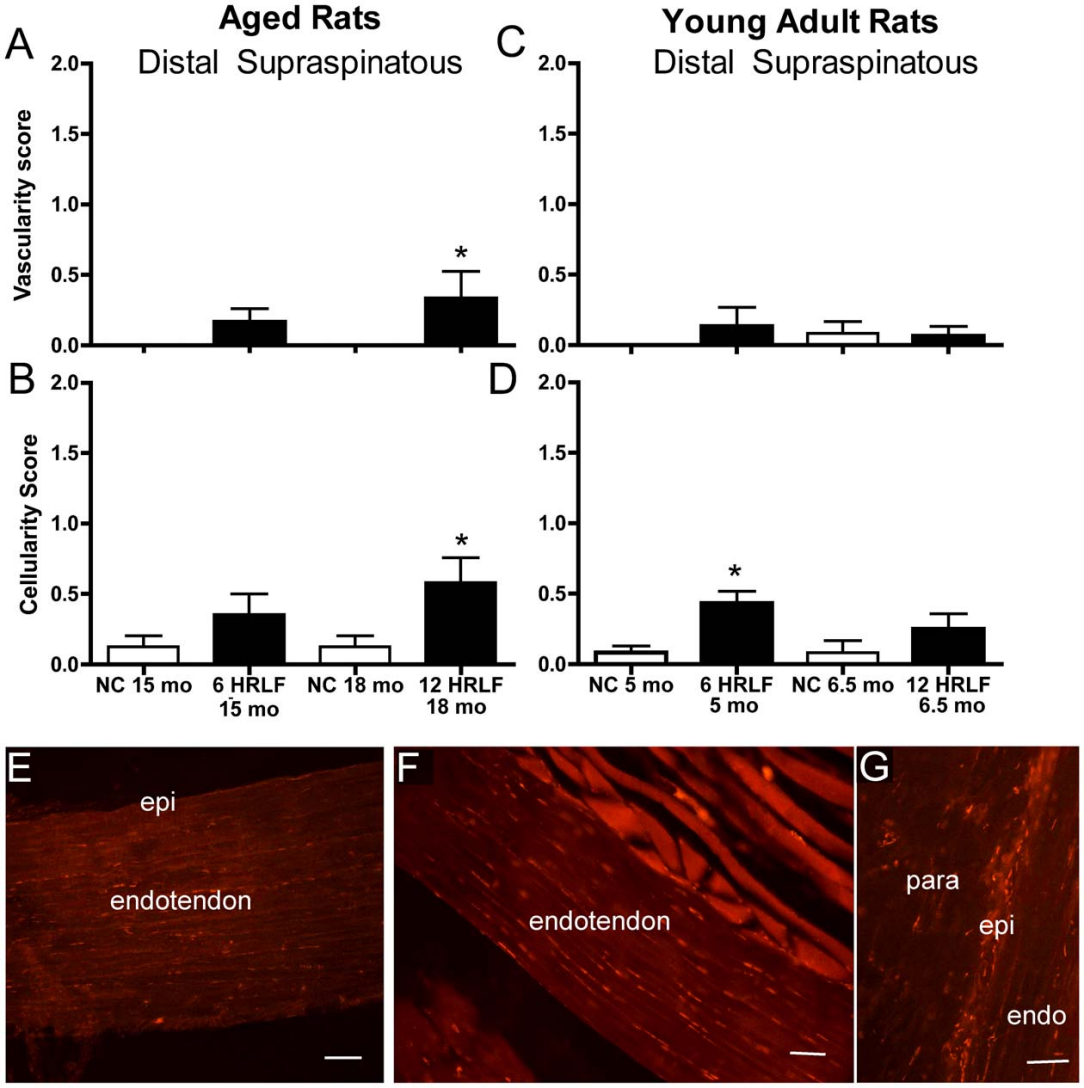
Histomorphometry and CTGF immunoreactivity in distal supraspinatus tendons. *(A)* Cellularity scores of aged high repetition low force (HRLF) and aged normal control (NC) rat tendons. *(B)* Cellularity scores in young adult rat tendons. *p<0.05, compared to NC. *(C)* CTGF in distal supraspinatus tendon of aged NC, and *(D)* aged 12-week HRLF rat. *(E)* CTGF in distal supraspinatus epitendon of aged 12-week HRLF rat. Epi = epitendon, endo = endotendon; para = paratendon. Scale bar = 50 µm.

When quantified with the Bonar scale, there were also no changes in collagen organization in supraspinatus tendons of either age group, compared to age-matched NC (Fig. 4,5), nor was there evidence of calcification in supraspinatus tendons (Fig. 5). In contrast, when quantified with the Bonar scale, vascularity increased in distal supraspinatus tendons of aged 12-week HRLF rats, compared to NC (p<0.05; Fig. 5A), although not in young adult HRLF rats (Fig. 5B). Also when quantified with the Bonar scale, cellularity increased in the enthesis and distal supraspinatus tendons of aged 12-week HRLF rats, compared to age-matched NC (Fig. 4C,5C). Cellularity also increased significantly by week 6 in distal supraspinatus tendons of young adult HRLF rats, compared to NC (p<0.05; Fig. 5D). This increase had resolved by week 12 (Fig. 4G,H).

When cellularity was quantified with a bioquantification image analysis system, we found that CTGF-immunoreactivity (-IR) increased in distal epitendon of the supraspinatus of aged 12-week HRLF rats, compared to age-matched NC (p<0.05, aged 12 wk HRLF = 8.89+2.98 (Mean + SEM), compared to aged NC = 1.821+0.57; Fig. 5E–G for photos). The CTGF-IR cell profiles appeared to be fibroblasts and endothelial cells. CTGF-IR cell profiles did not increase in supraspinatus endotendons (Fig. 5E,F). Similar results were observed in flexor digitorum tendons of both age groups (data not shown).

**Figure 6:**
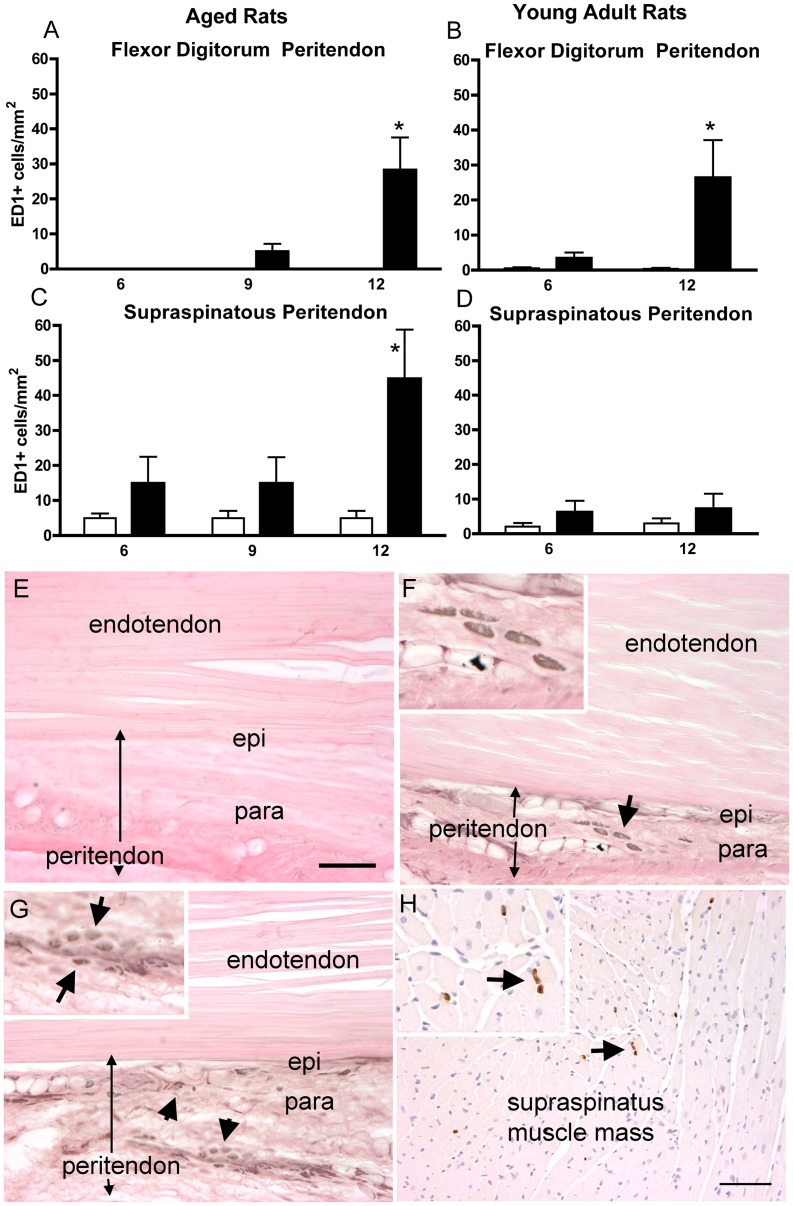
ED1-immunoreactive macrophages in flexor digitorum and supraspinatus peritendon regions of preferred reach limbs. *(A)* ED1 cells in the flexor digitorum peritendon in aged rats, and *(B)* in young adult rats. *(C)* ED1 cells in supraspinatus endotendon of aged rats, and *(D)* in young adult rats. *p<0.05, compared to age-matched NC. *(E)* Photo of a flexor digitorum tendon of an aged-NC rat, and *(F)* in an aged 12-week HRLF rat. Arrow depicts ED1 cells in paratendon (ED1 is black; eosin counterstain), shown enlarged in the inset. *(G)* Photo of a distal cut end of a supraspinatus tendon of an aged 12-week HRLF rat. Arrows depict ED1 cells in paratendon, shown enlarged in inset. *(H)* Photo of the muscle mass of an aged 12-week HRLF rat showing ED1 cells. Arrow depicts ED1 cells (brown in color; eosin counterstain), shown enlarged in inset. Scale bar = 50 micrometers.

Also, when quantified with a bioquantification system, ED1-IR cells (activated macrophages) increased in flexor digitorum peritendons of aged and young adult 12-week HRLF rats, compared to age-matched NC (p<0.05 post hoc; Fig. 6A, B, E, F). ED1-IR cells were located in the peritendon, but not the epitendon, of aged 12-week HRLF rats (Fig. 6F) or the endotendon region of the flexor digitorum of either age group (data not shown). Furthermore, ED1-IR cells increased in supraspinatus peritendons of aged 12-week HRLF rats, compared to age-matched NC (Fig. 6C,G), but not in young adult HRLF rats (Fig. 6D). ED1-IR cells did not increase in the supraspinatus endotendon of either age group (Fig. 6G; other data not shown). Although not quantified, increased ED1-IR cells were also observed in the supraspinatus muscle mass of aged 12-week HRLF rats (Fig. 6H).

## Discussion

Performance of a HRLF task for 3 months resulted in an increase in the percentage of successful reaches in young adult rats, but not in aged rats. There was also a persistent decrease in forelimb agility in the aged 12-week HRLF rats. HRLF task performance increased IL-1beta and IL-6 levels in the flexor digitorum tendons of aged rats, but not young adult rats. In contrast, although TNF-alpha levels were higher in aged task rats than aged NC rats, TNF-alpha levels were even higher in young adult task rats. Cellularity increased in the flexor digitorum tendons of both age groups by week 12 of HRLF performance, but only in the supraspinatus tendons of aged HRLF rats by week 12. This increase in cellularity was due to increased ED1-IR macrophages and CTGF-IR cells, the latter presumably fibroblasts and endothelial cells, since CTGF is known to be present in each [33], [35], [42]. The greater inflammatory changes in aged HRLF rat tendons temporally matched the decreased forelimb agility.

We recently reported an increase in the percent successful reaches in young adult rats performing the HRLF task for 12 weeks [9]. What was surprising was not to see the same increase in aged 12-week HRLF rats. Perhaps the aged rats were not able to learn the HRLF task as well as young adult rats. It is also possible that the increased IL-1beta and IL-6 in flexor digitorum tendons and macrophages in supraspinatus tendons of aged HRLF rats contributed to discomfort that prevented task improvement. The persistent decrease in ability to perform the forehead sticker removal test by aged HRLF rats at matched time points as the increased IL-6 in the flexor digitorum tendons and ED1-IR macrophages in supraspinatus tendons further supports this hypothesis. We have shown that ibuprofen treatment of rats performing a high repetition high force (HRHF) handle-pulling task significantly improves reach rate, reach force, and duration [9]; this also supports an strong inflammatory influence on motor performance.

Several pro-inflammatory cytokines increased significantly in flexor digitorum tendons of aged task rats (IL-1beta, TNFalpha, and IL-6 ), compared to aged NC rats. TNFalpha were also higher in these tendons of young adult 12-week HRLF rats (2.5-fold), than in young adult NC rats. Therefore, in each age group, cytokines increased in tendons with HRLF performance. Other labs have also observed increased inflammatory cytokines in tendons with overuse, in association with carpal tunnel syndrome, or after prolonged exercise, including increased IL-1beta and IL-6 [43], [44], [45], [46], [47], [48], [49]. We also noted greater increases of IL-1beta and IL-6 in aged task rats, than in young task rats. The level of their increases were similar to those observed in young rats performing higher demand tasks, such as a high repetition high force (HRHF) task and a moderate repetition high force (MRHF) handle-pulling task [35] [50], for 12 weeks. This suggests that the combination of aging and HRLF task performance leads to greater responses of IL-1beta and IL6, than in young rats performing the same task.

We have previously observed large increases in serum levels of several inflammatory cytokines (IL-1alpha, IL-6, and interferon gamma) in aged rats in general, compared to young adult rats [13]. Serum IL-6 increased even further in aged rats performing the HRLF task for 12 weeks. These results match those of others showing that serum and tissue levels of several inflammatory cytokines increase with aged patients and animals lacking any apparent illness [51], [52]. People have hypothesized that these increases are due to altered regulation of the production of these cytokines with aging [53], [54]. A rise in inflammatory cytokines with aging combined with increases occurring as a consequence repetitive work tasks may render an individual and their tissues susceptible to a myriad of processes induced by these cytokines, including increased catabolic processes [55], [56].

We also observed increased cellularity in flexor digitorum peritendons of both aged and young adults rat performing the HRLF task for 12 weeks, and in the supraspinatus tendons of aged rats. The increase in peritendon cellularity was due to increased in CTGF-IR and ED1-IR cell profiles. This matches previously reported results from our lab showing increased fibrotic and inflammatory changes in peritendons of rats performing HRHF tasks [10], [35], [57], [58]. The task-induced fibrotic changes observed in this study were considerably less than in that study, indicating that high force tasks drive tendon fibrotic changes more than low force tasks. The variability in presence or absence of these fibrotic changes in the HRLF rats in this study (See Fig. 5D,E), further supports this hypothesis. The increase in ED1-IR cells in peritendon tissues is similar to studies examining tenosynovium from patients with carpal tunnel syndrome in which inflammatory changes (as well as fibrotic changes) have been observed [59], [60].

Lastly, we found no increase of ED1-IR cells or collagen fibril disorganization in the endotendons in this study. This contrasts to prior studies from our lab showing increased ED1-IR macrophages and collagen disorganization in the endotendon region of flexor digitorum tendons of rats performing a HRHF task [35]. Prolonged downhill running has also been shown to induce visible collagen disorganization in flexor digitorum and supraspinatus tendons [61], [62]. These results combined indicate that the HRLF task, does not load upper extremity tendons as much as a HRHF task or downhill running, and is not as tissue damaging. This is evidence of an exposure-response relationship that is both force- and rate-dependent and which is consistent with epidemiological findings concerning WMSDs.

There are several limitations in this study. The use of female rats only precludes the generalization of our findings to males. However, since we scaled the force to the average maximum pulling force of adult female rats, we hypothesize that the exposure-dependent tissue and behavior responses would be similar in males. Since WMSDs of the wrist and hand are more prevalent among females, we deemed the use of female rats to be appropriate for this study. Next, the young adult HRLF rats were not euthanized for ELISA analysis until week 12 of task performance. Therefore, tissues from prior weeks were not available for cytokine analysis. Nor did we analyze young adult HRLF rats for histomorphological changes at time points other than 6 or 12 weeks after onset of task performance. This allowed us to compare tendon changes in aged versus young adult rats at only two endpoints, 6 and 12 weeks. We also focused on tendon changes only here. Although we have previously reported changes in muscle, bone, nerve, and more in our model [10], [33], [38], [39], [41], [42], [50], [57], [63], this focus on tendons allowed us to explore its changes more thoroughly than if all tissues had been included for examination.

In conclusion, flexor digitorum tendons were less affected by this HRLF task than in our prior studies examining the effects of high force tasks. Aged rat tendons were more affected by this HRLF task than young adult, particularly in supraspinatus tendons. The greater inflammatory changes in aged HRLF rat tendons temporally correlated with the decreased forelimb agility and lack of improvement in task success. These data support prior reports of a decrease in motor function as a consequence of repetitive work tasks. This decrease was related to increased inflammatory responses in the aged rat tendons rather than a greater propensity for injury or degenerative processes.

## References

[1] Bureau of Labor Statistics website. (2011) Nonfatal ocupational injuries and illnesses requiring days away from work, 2010. Available: http://www.bls.gov/news.release/osh2.nr0.htm. Accessed: 2012 Sept 11.

[2] Kilbom S, Armstrong TJ, Buckle P, Fine L, Hagberg M, et al (1996) Musculoskeletal Disorders: Work-related Risk Factors and Prevention. International Journal of Occupational and Environmental Health 2: 239–246. 9933878 10.1179/oeh.1996.2.3.239

[3] Bernard BP (1997) Musculoskeletal Disorders and Workplace Factors: A Critical Review of Epidemiologic Evidence for Work-Related Musculoskeletal Disorders of the Neck, Upper Extremity, and Low Back. US Department of Health and Human Services.

[4] Gerr F, Marcus M, Ensor C, Kleinbaum D, Cohen S, et al (2002) A prospective study of computer users: I. Study design and incidence of musculoskeletal symptoms and disorders. American Journal of Industrial Medicine 41: 221–235. 11920966 10.1002/ajim.10066

[5] Nunes IL (2009) FAST ERGO_X - a tool for ergonomic auditing and work-related musculoskeletal disorders prevention. Work 34: 133–148. 20037227 10.3233/WOR-2009-0912

[6] McDermott HJ, Kazi A, Munir F, Haslam C (2010) Developing occupational health services for active age management. Occupational medicine 60: 193–204. 20423950 10.1093/occmed/kqq026

[7] Akrouf QA, Crawford JO, Al-Shatti AS, Kamel MI (2010) Musculoskeletal disorders among bank office workers in Kuwait. Eastern Mediterranean health journal = La revue de sante de la Mediterranee orientale = al-Majallah al-sihhiyah li-sharq al-mutawassit 16: 94–100. 20214165

[8] Carp SJ, Barbe MF, Winter KA, Amin M, Barr AE (2007) Inflammatory biomarkers increase with severity of upper-extremity overuse disorders. Clinical Science 112: 305–314. 17064252 10.1042/CS20060050

[9] Kietrys DM, Barr AE, Barbe MF (2011) Exposure to repetitive tasks induces motor changes related to skill acquisition and inflammation in rats. Journal of Motor Behavior 43: 465–476. 22087754 10.1080/00222895.2011.627897 PMC3727635

[10] Rani S, Barbe MF, Barr AE, Litivn J (2010) Role of TNF alpha and PLF in bone remodeling in a rat model of repetitive reaching and grasping. Journal of cellular physiology 225: 152–167. 20458732 10.1002/jcp.22208 PMC3688633

[11] Rechardt M, Shiri R, Matikainen S, Viikari-Juntura E, Karppinen J, et al (2011) Soluble IL-1RII and IL-18 are associated with incipient upper extremity soft tissue disorders. Cytokine 54: 149–153. 21371906 10.1016/j.cyto.2011.02.003

[12] Health and Safety Executive Laboratory web site. Aging and work-related musculoskeletal disorders. Research Report RR799. Available: http://www.hse.gov.uk/research/rrhtm/rr799.htm. Accessed: 2012 Sept 11.

[13] Xin DL, Harris MY, Wade CK, Amin M, Barr AE, et al (2011) Aging enhances serum cytokine response but not task-induced grip strength declines in a rat model of work-related musculoskeletal disorders. BMC Musculoskeletal Disorders 12: 63. 21447183 10.1186/1471-2474-12-63 PMC3072947

[14] Elliott MB, Barr AE, Clark BD, Wade CK, Barbe MF (2010) Performance of a repetitive task by aged rats leads to median neuropathy and spinal cord inflammation with associated sensorimotor declines. Neuroscience 170: 929–941. 20673790 10.1016/j.neuroscience.2010.07.041

[15] van Rijn RM, Huisstede BM, Koes BW, Burdorf A (2010) Associations between work-related factors and specific disorders of the shoulder–a systematic review of the literature. Scandinavian Journal of Work, Environment & Health 36: 189–201. 10.5271/sjweh.2895 20094690

[16] Miranda H, Punnett L, Viikari-Juntura E, Heliovaara M, Knekt P (2008) Physical work and chronic shoulder disorder. Results of a prospective population-based study. Annals of the Rheumatic Diseases 67: 218–223. 17526553 10.1136/ard.2007.069419

[17] Ranney D, Well R, Moore A (1995) Upper limb musculoskeletal disorders in highly repetitive industries: precise anatomical physical findings. Ergonomics 38: 1408–1423. 7635130 10.1080/00140139508925198

[18] Bystrom S, Hall C, Welander T, Kilbom A (1995) Clinical disorders and pressure-pain threshold of the forearm and hand among automobile assembly line workers. Journal of Hand Surgery - British Volume 20: 782–790. 10.1016/s0266-7681(95)80047-6 8770741

[19] Lee MW, Park SC, Kim JH, Kim IK, Han KS, et al (2002) The involvement of oxidative stress in tumor necrosis factor related apoptosis-inducing ligand (TRAIL)-induced apoptosis in HeLa cells. Cancer Letters 182: 75–82. 12175526 10.1016/s0304-3835(02)00074-5

[20] Millar NL, Wei AQ, Molloy TJ, Bonar F, Murrell GA, et al (2009) Cytokines and apoptosis in supraspinatus tendinopathy. Journal of Bone & Joint Surgery - British Volume 91: 417–424. 10.1302/0301-620X.91B3.21652 19258623

[21] Voloshin I, Gelnas J, Maloney MD, OKeefe RJ, Bigliani LU, et al (2005) Proinflammatory cytokines and metalloproteases are expressed in the subacromial bursa in patients with rotator cuff disease. Arthroscopy 21: 1076. 16171632 10.1016/j.arthro.2005.05.017

[22] Archambault JM, Jelinsky SA, Lake SP, Hill AA, Glaser DL, et al (2007) Rat supraspinatus tendon expresses cartilage markers with overuse. Journal of Orthopaedic Research 25: 617–624. 17318892 10.1002/jor.20347

[23] Carpenter JE, Flanagan CL, Thomopoulos S, Yian EH, Soslowsky LJ (1998) The effects of overuse combined with intrinsic or extrinsic alterations in an animal model of rotator cuff tendinosis. American Journal of Sports Medicine 26: 801–807. 9850782 10.1177/03635465980260061101

[24] Millar NL, Wei AQ, Molloy TJ, Bonar F, Murrell GA, et al (2008) Heat shock protein and apoptosis in supraspinatus tendinopathy. Clinical Orthopaedics & Related Research 466: 1569–1576. 18459030 10.1007/s11999-008-0265-9 PMC2505259

[25] Perry SM, McIlhenny SE, Hoffman MC, Soslowsky LJ (2005) Inflammatory and angiogenic mRNA levels are altered in a supraspinatus tendon overuse animal model. Journal of Shoulder & Elbow Surgery 14: 79S–83S. 15726091 10.1016/j.jse.2004.09.020

[26] Soslowsky LJ, Thomopoulos S, Esmail A, Flanagan CL, Iannotti JP, et al (2002) Rotator cuff tendinosis in an animal model: role of extrinsic and overuse factors. Annals of Biomedical Engineering 30: 1057–1063. 12449766 10.1114/1.1509765

[27] Soslowsky LJ, Thomopoulos S, Tun S, Flanagan CL, Keefer CC, et al (2000) Neer Award 1999. Overuse activity injures the supraspinatus tendon in an animal model: a histologic and biomechanical study. Journal of Shoulder & Elbow Surgery 9: 79–84. 10810684

[28] Szomor ZL, Appleyard RC, Murrell GAC (2006) Overexpression of nitric oxide synthases in tendon overuse. Journal of Orthopaedic Research 24: 80–86. 16419972 10.1002/jor.20009

[29] Brewer JB (1979) Aging in the rotator cuff. Am J Sports Med 7: 102–110. 434288 10.1177/036354657900700206

[30] Hsu JC, Luo ZP, Stone JJ, Huang TH, An KN (2003) Correlation between rotator cuff tear and glenohumeral degeration. Acta Orthopaedica Scandinavica 74: 89–94. 12635800 10.1080/00016470310013725

[31] Panni AS, Milano G, Lucania L, Fabbriciani C, Logroscino CA (1996) Histological analysis of the coracoacromial arch: correlation between age related changes and rotator cuff tears. Arthroscopy 12: 531–540. 8902125 10.1016/s0749-8063(96)90190-5

[32] Gerr F, Marcus M, Ensor C, Kleinbaum D, Cohen S, et al (2002) A prospective study of computer users: I. Study design and incidence of musculoskeletal symptoms and disorders. American Journal of Industrial Medicine 41: 221–235. 11920966 10.1002/ajim.10066

[33] Clark BD, Al-Shatti TA, Barr AE, Amin M, Barbe MF (2004) Performance of a high-repetition, high-force task induces carpal tunnel syndrome in rats. Journal of Orthopaedic & Sports Physical Therapy 34: 244–253. 15189016 10.2519/jospt.2004.34.5.244

[34] Driban JB, Barr AE, Amin M, Sitler MR, Barbe MF (2011) Joint inflammation and early degeneration induced by high-force reaching are attenuated by ibuprofen in an animal model of work-related musculoskeletal disorder. Journal of Biomedicine & Biotechnology 2011: 691412. 21403884 10.1155/2011/691412 PMC3051200

[35] Fedorczyk JM, Barr AE, Rani S, Gao HG, Amin M, et al.. (2010) Exposure-dependent increases in IL-1beta, substance P, CTGF, and tendinosis in flexor digitorum tendons with upper extremity repetitive strain injury. Journal of Orthopaedic Research. 10.1002/jor.20984 PMC2807907 19743505

[36] Coq JO, Barr AE, Strata F, Russier M, Kietrys DM, et al.. (2009) Peripheral and central changes combine to induce motor behavrioal declines in a moderate repetition task. Experimental Neurology, in press. 10.1016/j.expneurol.2009.08.008 PMC2783426 19686738

[37] Schrimsher GW, Reier PJ (1992) Forelimb motor performance following cervical spinal cord contusion injury in the rat. Experimental Neurology 117: 287–298. 1397165 10.1016/0014-4886(92)90138-g

[38] Barbe MF, Elliott MB, Abdelmagid SM, Amin M, Popoff SN, et al (2008) Serum and tissue cytokines and chemokines increase with repetitive upper extremity tasks. Journal of Orthopaedic Research 26: 1–11. 18464247 10.1002/jor.20674

[39] Barr AE, Safadi FF, Gorzelany I, Amin M, Popoff SN, et al (2003) Repetitive, negligible force reaching in rats induces pathological overloading of upper extremity bones. Journal of Bone & Mineral Research 18: 2023–2032. 14606516 10.1359/jbmr.2003.18.11.2023

[40] Cook JL, Feller JA, Bonar SF, Khan KM (2004) Abnormal tenocyte morphology is more prevalent than collagen disruption in asymptomatic athletes' patellar tendons. Journal of Orthopaedic Research 22: 334–338. 15013093 10.1016/j.orthres.2003.08.005

[41] Barbe MF, Barr AE, Gorzelany I, Amin M, Gaughan JP, et al (2003) Chronic repetitive reaching and grasping results in decreased motor performance and widespread tissue responses in a rat model of MSD. Journal of Orthopaedic Research 21: 167–176. 12507595 10.1016/S0736-0266(02)00086-4 PMC1560095

[42] Clark BD, Barr AE, Safadi FF, Beitman L, Al-Shatti T, et al (2003) Median nerve trauma in a rat model of work-related musculoskeletal disorder. Journal of Neurotrauma 20: 681–695. 12908929 10.1089/089771503322144590 PMC1550513

[43] Sun HB, Li Y, Fung DT, Majeska RJ, Schaffler MB, et al (2008) Coordinate regulation of IL-1beta and MMP-13 in rat tendons following subrupture fatigue damage. Clinical orthopaedics and related research 466: 1555–1561. 18470577 10.1007/s11999-008-0278-4 PMC2505236

[44] Berglund M, Hart DA, Wiig M (2007) The inflammatory response and hyaluronan synthases in the rabbit flexor tendon and tendon sheath following injury. The Journal of hand surgery, European volume 32: 581–587. 10.1016/J.JHSE.2007.05.017 17950228

[45] Asundi KR, Rempel DM (2008) MMP-1, IL-1beta, and COX-2 mRNA expression is modulated by static load in rabbit flexor tendons. Annals of Biomedical Engineering 36: 237–243. 18172766 10.1007/s10439-007-9427-2

[46] Tucci MA, Barbieri RA, Freeland AE (1997) Biochemical and histological analysis of the flexor tenosynovium in patients with carpal tunnel syndrome. Biomedical sciences instrumentation 33: 246–251. 9731366

[47] Langberg H, Olesen JL, Gemmer C, Kjaer M (2002) Substantial elevation of interleukin-6 concentration in peritendinous tissue, in contrast to muscle, following prolonged exercise in humans. The Journal of physiology 542: 985–990. 12154195 10.1113/jphysiol.2002.019141 PMC2290459

[48] Freeland AE, Tucci MA, Barbieri RA, Angel MF, Nick TG (2002) Biochemical evaluation of serum and flexor tenosynovium in carpal tunnel syndrome. Microsurgery 22: 378–385. 12497576 10.1002/micr.10065

[49] Freeland AE, McAuliffe JA (2002) Dorsal carpal metacarpal fracture dislocation associated with nondissociative volar intercalated segmental instability. Orthopedics 25: 753–755. 12138962 10.3928/0147-7447-20020701-16

[50] Elliott MB, Barr AE, Clark BD, Amin M, Amin S, et al (2009) High force reaching task induces widespread inflammation, increased spinal cord neurochemicals and neuropathic pain. Neuroscience 158: 922–931. 19032977 10.1016/j.neuroscience.2008.10.050 PMC2661572

[51] Ershler WB, Keller ET (2000) Age-associated increased interleukin-6 gene expression, late-life diseases, and frailty. Annual review of medicine 51: 245–270. 10.1146/annurev.med.51.1.245 10774463

[52] Fagiolo U, Cossarizza A, Scala E, Fanales-Belasio E, Ortolani C, et al (1993) Increased cytokine production in mononuclear cells of healthy elderly people. European journal of immunology 23: 2375–2378. 8370415 10.1002/eji.1830230950

[53] Daynes RA, Araneo BA, Ershler WB, Maloney C, Li GZ, et al (1993) Altered regulation of IL-6 production with normal aging. Possible linkage to the age-associated decline in dehydroepiandrosterone and its sulfated derivative. Journal of immunology 150: 5219–5230. 8515056

[54] Belmin J, Bernard C, Corman B, Merval R, Esposito B, et al (1995) Increased production of tumor necrosis factor and interleukin-6 by arterial wall of aged rats. The American journal of physiology 268: H2288–2293. 7611479 10.1152/ajpheart.1995.268.6.H2288

[55] Ershler WB, Sun WH, Binkley N, Gravenstein S, Volk MJ, et al (1993) Interleukin-6 and aging: blood levels and mononuclear cell production increase with advancing age and in vitro production is modifiable by dietary restriction. Lymphokine and cytokine research 12: 225–230. 8218595

[56] Roubenoff R, Parise H, Payette HA, Abad LW, D'Agostino R, et al (2003) Cytokines, insulin-like growth factor 1, sarcopenia, and mortality in very old community-dwelling men and women: the Framingham Heart Study. The American journal of medicine 115: 429–435. 14563498 10.1016/j.amjmed.2003.05.001

[57] Rani S, Barbe MF, Litvin J (2009) Induction of Periostin-like Factor and Periostin in Forearm Muscle, Tendon, and Nerve in an Animal Model of Work-related Musculoskeletal Disorder. Journal of Histochemistry & Cytochemistry. 10.1369/jhc.2009.954081 PMC2762884 19620321

[58] Abdelmagid SA, Rickard JA, McDonald WJ, Thomas LN, Too CK (2011) CAT-1-mediated arginine uptake and regulation of nitric oxide synthases for the survival of human breast cancer cell lines. Journal of cellular biochemistry 112: 1084–1092. 21308737 10.1002/jcb.23022

[59] Nakamichi K, Tachibana S (1998) Histology of the transverse carpal ligament and flexor tenosynovium in idiopathic carpal tunnel syndrome. The Journal of hand surgery 23: 1015–1024. 9848552 10.1016/s0363-5023(98)80009-9

[60] Schuind F, Ventura M, Pasteels JL (1990) Idiopathic carpal tunnel syndrome: histologic study of flexor tendon synovium. The Journal of hand surgery 15: 497–503. 2348074 10.1016/0363-5023(90)90070-8

[61] Scott A, Cook JL, Hart DA, Walker DC, Duronio V, et al (2007) Tenocyte responses to mechanical loading in vivo: a role for local insulin-like growth factor 1 signaling in early tendinosis in rats. Arthritis and rheumatism 56: 871–881. 17328060 10.1002/art.22426

[62] Soslowsky LJ, Thomopoulos S, Tun S, Flanagan CL, Keefer CC, et al (2000) Neer Award 1999. Overuse activity injures the supraspinatus tendon in an animal model: a histologic and biomechanical study. Journal of shoulder and elbow surgery/American Shoulder and Elbow Surgeons [et al] 9: 79–84. 10810684

[63] Rani S, Barbe MF, Barr AE, Litvin J (2009) Periostin-like-factor and Periostin in an animal model of work-related musculoskeletal disorder. Bone 44: 502–512. 19095091 10.1016/j.bone.2008.11.012 PMC3730819

